# Age-dependent immune responses in COVID-19-mediated liver injury: focus on cytokines

**DOI:** 10.3389/fendo.2023.1139692

**Published:** 2023-08-15

**Authors:** Nazanin Aghamohamadi, Faezeh Shahba, Ali Zarezadeh Mehrabadi, Hossein Khorramdelazad, Milad Karimi, Reza Falak, Reza Zolfaghari Emameh

**Affiliations:** ^1^ Department of Immunology, School of Medicine, Iran University of Medical Sciences, Tehran, Iran; ^2^ Department of Immunology, School of Medicine, Rafsanjan University of Medical Sciences, Rafsanjan, Iran; ^3^ Department of Energy and Environmental Biotechnology, National Institute of Genetic Engineering and Biotechnology (NIGEB), Tehran, Iran

**Keywords:** COVID-19, SARS-CoV-2, cytokine storm, interleukin, liver injury, aging

## Abstract

Severe acute respiratory syndrome coronavirus 2 (SARS-CoV-2) is potentially pathogenic and causes severe symptoms; in addition to respiratory syndromes, patients might experience other severe conditions such as digestive complications and liver complications injury. The abnormality in the liver is manifested by hepatobiliary dysfunction and enzymatic elevation, which is associated with morbidity and mortality. The direct cytopathic effect, immune dysfunction, cytokine storm, and adverse effects of therapeutic regimens have a crucial role in the severity of liver injury. According to aging and immune system alterations, cytokine patterns may also change in the elderly. Moreover, hyperproduction of cytokines in the inflammatory response to SARS-CoV-2 can lead to multi-organ dysfunction. The mortality rate in elderly patients, particularly those with other comorbidities, is also higher than in adults. Although the pathogenic effect of SARS-CoV-2 on the liver has been widely studied, the impact of age and immune-mediated responses at different ages remain unclear. This review discusses the association between immune system responses in coronavirus disease 2019 (COVID-19) patients of different ages and liver injury, focusing on cytokine alterations.

## 1 Introduction

The coronaviridae family comprises enveloped viruses infecting amphibians, birds, and mammals. The family consists of the torovirinae and orthocoronavirinae; the subfamily orthocoronavirinae includes alpha, beta, gamma, and deltacoronaviruses (1–4). Coronaviruses have crown-like structures due to spike glycoproteins. These viruses have an outer envelope and contain positive-stranded RNA as their genomic material (5).

Some species of coronaviruses may not lead to severe disease and are typically responsible for common colds. However, severe acute respiratory syndrome coronavirus (SARS-CoV), Middle East respiratory syndrome coronavirus (MERS-CoV), and SARS-CoV-2 are potentially pathogenic to humans. These viruses can cause severe respiratory complications, multiple organ failure, and even death in severe stages (6, 7). SARS-CoV-2, the last known species of the coronavirus family, is a beta-coronavirus and was identified in Wuhan Hubei Province, China, in late 2019 (8). The COVID-19 clinical manifestations vary from sore throat, cough, fever, and loss of smell or taste to acute respiratory distress syndrome (ARDS), leading to patient hospitalization (9). Moreover, due to SARS-CoV-2-induced thrombotic disorders, various organ-specific complications have been observed, including heart injury, acute kidney injury, liver dysfunction, central nervous system (CNS) complications, and gastroenteritis (9, 10).

According to the lessons from patients with SARS-CoV-1, various levels of hepatopathy and elevated liver enzymes suggest that coronaviruses are responsible for inducing systemic inflammation (11–13). In a similar behavior, SARS-CoV-2 can also increase aminotransferase levels (14).

Several hepatotoxic medications, particularly those used to treat COVID-19, have been related to drug-induced liver damage (15). However, liver injury is defined as any damage to the liver that occurs during disease or treatment (16). As a result, the proportion of hospitalized COVID-19 patients with altered liver biomarkers ranges from 14% to 53%. Evidence has revealed that the levels of aminotransferase, bilirubin, alkaline phosphatase (ALP), and gamma-glutamyl transferase (GGT) are remarkably increased in patients with COVID-19 (17, 18). In contrast, serum albumin levels decrease in SARS-CoV-2-induced liver injury (19).

Cytokine storm is characterized as an abnormal, excessive, and uncontrolled systemic inflammatory response associated with the hyperproduction of pro-inflammatory cytokines. This inflammatory phenomenon is caused by infection, drugs, or an allogeneic hypersensitivity to foreign tissue, resulting in multi-organ functional impairment (20). Among the affected body organs, the liver is more susceptible to disruptions in systemic homeostasis and is influenced by various mechanisms, including vascular and immune-mediated pathways (21). The pathophysiology of liver dysfunction caused by the cytokine storm is characterized by increased vascular permeability due to cytokine release, hypoperfusion, endothelial dysfunction, reactive oxygen species (ROS) production, and nitric oxide (NO) deficiency (22). Furthermore, dysregulated cytokine release can cause an acute-phase response in hepatocytes (23). Multiple studies have demonstrated that immune responses vary with age, and this is also true for COVID-19 patients and their related cytokine responses (24, 25). Additionally, aging can affect cytokine patterns and exacerbate inflammation (26). Since the tissue damage caused by the cytokine storm is critical, aging and increased inflammation caused by SARS-CoV-2 infection may lead to more severe liver damage in the elderly than in younger patients (27).

Therefore, this review summarizes the association of age-related immune responses and cytokine patterns with liver injuries during SARS-CoV-2 infection or following treatment and vaccination.

## 2 Aging and immune system

**Figure 1:**
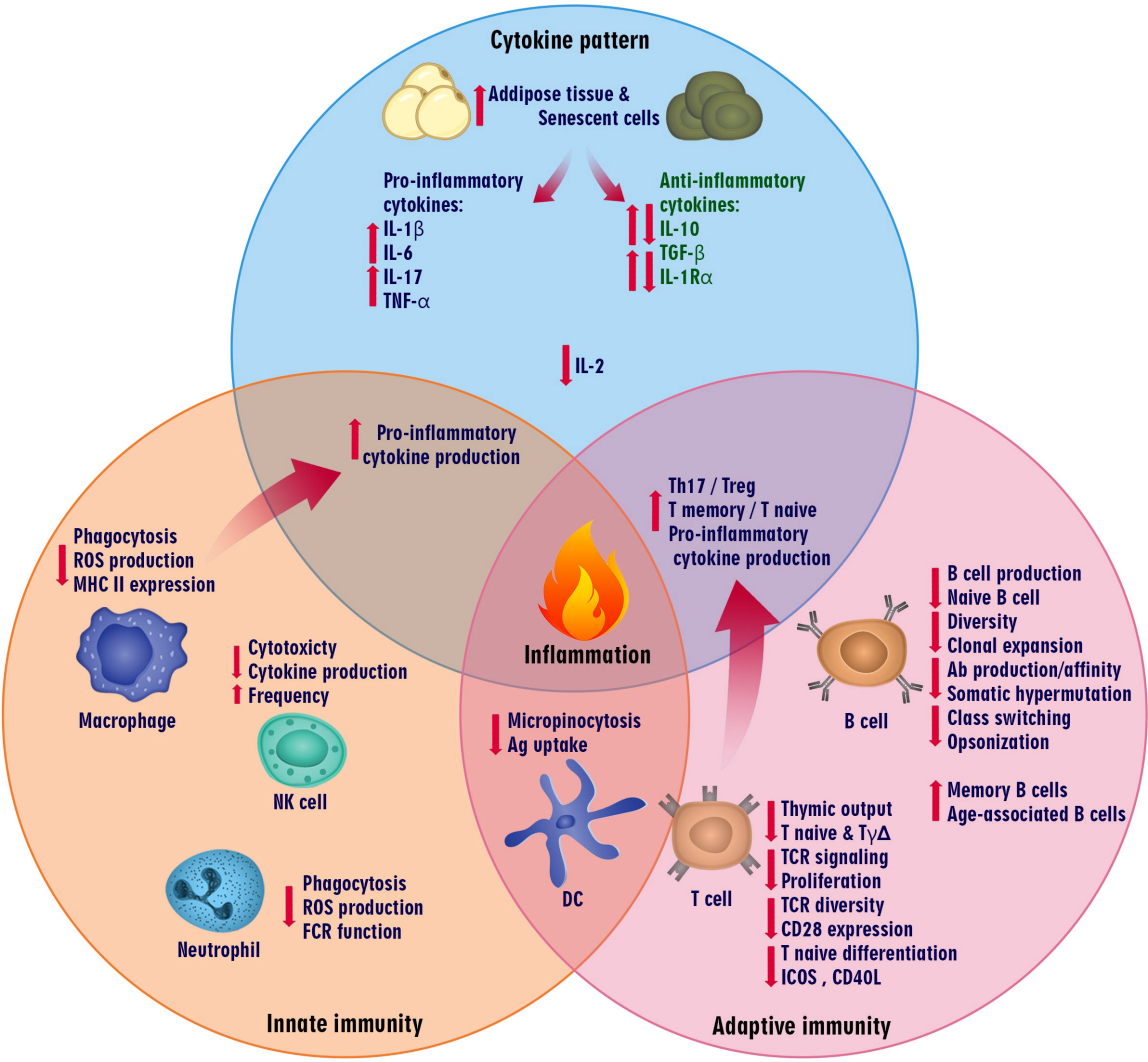
Immune system fluctuations in aging. The components of the innate and adaptive immune systems change in aging, affecting immune responses. These changes can also alter the cytokine pattern so that inflammatory conditions prevail in these people due to the increased expression of pro-inflammatory cytokines such as IL-1β, IL-6, IL-17, and TNF-α.

Aging is an intricate biological process associated with numerous alterations that can affect several organs’ functions and represents the leading risk factor for geriatric diseases (28). Age-related changes occur in every immune system component, called “immunosenescence,” and affect innate and adaptive immune responses (29, 30). These alterations in the immune system consist of persistent low-grade inflammation (called inflammaging), impaired ability to respond to new antigens effectively, and increased susceptibility to infections, autoimmunity, as well as cancer (**Figure 1**) (31, 32).

### 2.1 Innate immune system

Innate immunity is the first line of defense against pathogens and is vital for protecting against several infections (33). Previous studies have shown that aging impairs epithelial barriers in the skin, lung, and gastrointestinal tract, allowing pathogenic organisms to accumulate in mucosal tissues and challenge the innate immune system (34, 35). Immunosenescence affects innate immune cells such as neutrophils, macrophages, dendritic cells, and NK cells (36).

Studies evaluating the neutrophil phagocytosis of opsonized bacteria have revealed a considerable reduction in neutrophil phagocytic ability among the elderly population (37). Furthermore, macrophages exert their function by eliminating invasive pathogens and cancer cells by releasing pro-inflammatory cytokines, which can activate signals to other immune cells (38). Previous evidence has confirmed the harmful impact of aging on macrophage activity. The macrophage population expresses lower major histocompatibility complex (MHC) II in the elderly, decreasing the CD4^+^ T cell-mediated immune responses (39–41).

Moreover, it has been reported that macrophages could not produce ROS in aged rats after the interferon-gamma (IFN-γ) treatment (42). Reduced superoxide anions in macrophages may cause persistent infections in old people (42). In this way, studies on the micropinocytosis capacity of dendritic cells (DCs) have shown that aged DCs in the elderly are less able to uptake fluorescein isothiocyanate–dextran than young adult Mo-DCs (43). Unlike the other innate immune cells, the NK cell absolute count is elevated in the aged population (44). However, NK cell cytotoxicity and cytokine production can be reduced through aging (45). Aging also affects phagocytic and NK cells, soluble components, cytokines, and chemokines (46). Elevated serum concentrations of IL-1β, IL-6, and TNFα have been observed among elderly populations, leading to systemic inflammation via the continuous activation of various immune cells. Therefore, pro-inflammatory cytokines can be a prognostic indicator for functional impairment, weakness, morbidity, and mortality among the aged population (47, 48).

### 2.2 Adaptive immune system

The adaptive immune system is based on generating a diverse repertoire of T and B lymphocyte receptors (TCRs and BCRs), lymphocyte activation, and clonal expansion. The *de novo* generation of T and B cells has declined due to age-related alterations in the human hematopoietic system (29, 49). Hematopoietic stem cells (HSCs) are responsible for the continuous supply of myeloid and lymphoid progenitors; however, studies have shown that bone marrow (BM) cellularity and adaptive immune system functions decreased, whereas the frequency of HSC changes through aging (50–52). According to aging, the HSCs’ proliferative potency can decrease and shift toward myeloid progenitors (53, 54).

#### 2.2.1 B cell

In accordance with HSC diminished capacity, B lymphocyte quantitation and qualification have changed through aging. These age-related alterations include the decreased total number of B cells, diminished diversity of B cell repertoire, especially IgVh CDR3 diversity (55), declined naïve B cells frequency, impaired response capacity to new antigens (56), decreased memory B cells clonal expansion and plasma cells production (31, 50, 57). Besides a decline in antibody production, antibody functionality is also impaired by decreased affinities and opsonizing properties (31, 50, 57). As a result, the humoral immune response to extracellular antigens and vaccination is impaired during aging (31, 58).

Immunosenescence is characterized by significant changes in the naive/memory B cell compartment through a progressive shift from naïve B cells to memory B cells (29, 31, 58). Furthermore, several studies have introduced a subtype of B cells called age-associated B cells (ABCs) that are enhanced with age and express T-bet transcription factors and CD11c (59–61). The ABC subset does not proliferate after BCR crosslinking but exhibits a robust proliferation response after stimulation with toll-like receptor (TLR)-7 and TLR-9 ligands (61–63). Furthermore, ABCs are a pro-inflammatory subset that secretes significantly high amounts of TNF-α, impairing young Pro-B cell generation (64).

#### 2.2.2 T cell

Modifications in hematopoietic stem cells through aging play a crucial role in developing the T cell repertoire similar to B cells (31). The overall number of T cells, remarkably naïve CD8^+^ and γδTCR^+^ T cells, proliferation capacity of naïve T cells, TCR signaling, and diversity of TCRVβ repertoire are decreased in aging (49, 50, 65). In contrast, T memory/T naïve ratios, Th17/Treg ratios, and pro-inflammatory cytokine production is increased (29, 58, 66). Furthermore, thymo-suppressive cytokines such as IL-6, oncostatin M, and leukemia inhibitory factor increased, and thymo-stimulative cytokines such as IL-7 decreased, diminishing naïve T-cell output and increasing the susceptibility to infection, autoimmunity, and cancer (57, 58, 67).

Following the aging process, the co-stimulatory molecules such as CD28 are down-regulated in naïve T cells, reducing the differentiation of naïve T cells into central and effector memory T cells (50, 68). Moreover, an age-related Erk-TCR phosphorylation through the reduction in microRNA-181a of naive CD4^+^ T cells attenuated signaling in these cells (31, 69, 70). Besides, CD40L and inducible T-cell COStimulator (ICOS) expression decreased in CD4^+^ memory T cells, reducing B cell differentiation and specific antibody production (70).

### 2.3 The pattern of cytokines in aging

According to aging and the alterations of the immune system, it seems that cytokine patterns also change with aging. Inflammaging is a physiological process involving various organs and tissues, such as adipose tissue, and the accumulation of senescent cells in tissues (30, 57). It has been reported that inflammaging is a persistent low-grade inflammation associated with several age-related disorders. This phenomenon is characterized by increased levels of pro-inflammatory mediators (31, 50, 58). Evidence has revealed that adipose tissue could increase throughout the body in aging and act as an endocrine source of mediators such as acute-phase proteins, hormones, pro-inflammatory cytokines, and growth factors (58, 71, 72). In this regard, the levels of circulating pro-inflammatory mediators such as IL-1β, IL-6, TNF-α, and IFN-γ increase in both homeostatic situations and in response to infections in the elderly compared with adults (57, 73, 74). Although the cytokine pattern in aging is variable, the results of clinical studies in this field are contradictory. In contrast, experimental studies showed an age-related shift from type 1 cytokines (IL-2, IL-12, IFN-γ) to type 2 cytokines (IL-4, IL-6, IL-10) in animal models (75–77).

In a physiologic condition, the expression of IL-6 is undetectable in the serum of young individuals. However, IL-6 levels can be increased by aging, which has been called “a cytokine for gerontologists” (73, 78, 79). Another age-associated pro-inflammatory cytokine is TNF-α, which can be increased in elderly individuals compared with young people, leading to the progression of age-related diseases (80–82). Increased TNF-α and IL-6 are also associated with weakness, a significant decline in muscle strength, an increased risk of cardiovascular and cerebrovascular conditions, and cognitive disorders among the elderly (73, 79, 83). Controversial findings have also been reported on the role of IL-1 in the aging process. Some studies have shown an age-associated increase in the IL-1R antagonist (IL-1Rα) and no significant age-associated changes in IL-1 (73, 79, 84). Additionally, IFN-γ is essential in cell-mediated immune response and defense against intracellular pathogens and viruses. The expression of IFN-γ in CD8^+^ naïve, effector, and memory T cells increased in the elderly compared with the young population. However, the expression of IFN-γ in naïve, effector, and memory CD4^+^ T cells decreased in old comparison with young individuals (73, 79, 85). Generally, findings are contradictory about the role and levels of IFN-γ in aging (50, 75, 79). IL-2, the “T-cell growth factor (TCGF),” is critical in different immunological responses. Most studies reported an age-related decline in the production of IL-2 by peripheral blood mononuclear cells (PBMC) in the elderly compared to young people (50, 73, 75). In contrast, previous studies indicated that IL-2 intracellular expression might be upregulated in CD8^+^ T cells among elderly individuals (75, 85). Additionally, it was reported that IL-2 serum levels were not altered in the healthy-aged population compared to the young controls (86).

As an anti-inflammatory cytokine, IL-10 can repress pro-inflammatory cytokine production and cell proliferation (87). Furthermore, conflicting results have been observed for IL-10 in the elderly population. Most studies have revealed that IL-10 production decreases or might be unchanged through aging (75, 88, 89). However, some studies have reported that serum IL-10 levels and its production is increased during aging due to the attempt of the immune system to suppress the pro-inflammatory response and return the immune homeostasis (73, 79, 90). Transforming growth factor beta (TGF-β) is another anti-inflammatory cytokine that can be elevated in the serum levels of octogenarians and centenarians (73, 91, 92). However, it was reported that there are no significant differences in TGF-β levels between the elderly and young women (93). These findings indicate that the cytokine pattern can be sex-dependent in the elderly.

## 3 Immunopathogenesis of SARS-CoV-2 infection

Understanding the immunopathogenesis of infectious diseases is essential because investigating its various aspects and monitoring the immune system’s responses can lead to discovering diagnostic biomarkers and therapeutic targets. This section discusses the immunopathogenesis of SARS-CoV-2 infection and the destructive role of pro-inflammatory cytokines.

### 3.1 Immunopathogenesis of SARS-CoV-2 infection

Following the SARS-CoV-2 infection and virus entrance, angiotensin-converting enzyme 2 (ACE2)-expressing type II alveolar epithelial cells are the main target of the virus (94, 95). However, a wide range of other cells in different organs, such as cholangiocytes in the liver, can express ACE2 and become infected (96).

The pathogen-associated molecular pattern (PAMP) and danger-associated molecular pattern (DAMP) are identified by immune sensors such as TLR-3, TLR-7, retinoic acid-inducible gene I (RIG-I)-like receptors such as RIG-I, and melanoma differentiation-associated protein 5 (MDA5), initiating IFN-I downstream signals (97, 98). The ligation of IFNs-I to their receptors (IFNRs) activates Janus kinases (JAKs), signal transducer and activator of transcription 1 (STAT1) and STAT2, expressing interferon-stimulated genes (ISGs) and stimulating antiviral immune responses (99).

After releasing IFNs and other pro-inflammatory cytokines and chemokines, immune cells, including neutrophils and monocytes, are recruited to the site of infection. Moreover, the epithelial and fibroblast cells can release pro-inflammatory cytokines, including TNF-α, IL-1β, IL-6, monocyte infiltration factor (MIF), IL-12, TGF-β, IL-21, IL-23, and IL-27, contributing to cytokine storm as well as neutrophil/monocyte migration and activation. The infiltration of these immune cells is the cause of neutrophilia and monocytosis in patients with COVID-19 (13, 100). Cytokine storm is a hallmark of COVID-19 pathogenesis resulting from the secretion of excessive proinflammatory cytokines and chemokines, and it induces SARS-CoV-2-related complications, particularly ARDS and multi-organ failures.

Considering the increase of neutrophils and the decrease of lymphocytes in COVID-19 patients, the ratio of neutrophils to lymphocytes (N/L ratio) is a suitable biomarker for predicting the severity of the disease (101).

The first line of defense against SARS-CoV-2 is phagocytosis. Neutrophils try to prevent the virus from spreading via neutrophil extracellular trap (NET) formation and releasing neutrophilic elastase and myeloperoxidase (MPO) into the extracellular space. Accordingly, NETosis can damage the lung and other tissues. Therefore, neutrophils act as a double-edged sword in SARS-CoV-2 infection, and neutrophilia is usually associated with poor prognosis, especially in hospitalized patients (102).

Monocytes and macrophages are other immune cells that control viral infections. They sense and capture viral antigens and present them to CD8^+^ T cells. These activated T cells highly express IFN-γ and IL-2 to eradicate viral pathogens and recruit other anti-viral immune cells (103). However, in SARS-CoV-2 pathogenesis, monocytes alter their phenotype from CD14^++^CD16^–^ to CD14^++^CD16^++^ and their functionality from anti-inflammatory to inflammatory (104). Studies have shown that T and B cells cooperate in shifting monocyte phenotype. In this scenario, T cells can activate monocytes via expressing colony-stimulating factors 1 and 2 (CSF1 and CSF2) as ligands to their receptors on the monocytes and thus cause hyperinflammation (105).

Additionally, B cells secrete a large amount of IL-6, lymphotoxin beta (LTB), and lymphotoxin alpha (LTA), which bind to monocytes’ receptors. High concentrations of IL-6 can induce T cells to produce inflammatory chemokines and cytokines such as IFN-γ and IL-1β (106). Moreover, these interactions activate monocytes to induce cytokine hyperproduction and promote cytokine-induced tissue damage in patients infected with SARS-CoV-2 (107). Therefore, similar to neutrophils, monocyte, and macrophage inflammatory phenotype in SARS-CoV-2 pathogenesis raises the question of whether monocytes are friend or foe.

Lymphopenia as an immune abnormality is detected in 96.1% of severe COVID-19 patients, and its degree correlates with clinical outcomes (108). An investigation reported that recovered patients exhibited a slow increase in CD4^+^ and CD8^+^ T cell frequency compared to healthy individuals despite a decline in disease onset (109). Several reasons have been speculated for COVID-19 lymphopenia. SARS-CoV-2 infects lymphocytes through the ACE2 receptor and CD147 (ACE2-dependent and ACE2-independent routes), reducing the number of lymphocytes (110). Additionally, lymphoid organs such as the thymus and spleen are involved in lymphopenia. Coronavirus animal models have demonstrated alterations in the weight and cellularity of the thymus and reduction of the CD4^+^/CD8^+^ thymocytes (111).

Moreover, considering the rapid clinical recovery of blood lymphocytes in COVID-19 patients, lymphocyte sequestration in the lungs and the gastrointestinal tract has been proposed as a simple reason for lymphopenia (112). It has been suggested that T cells from COVID-19 patients undergo apoptosis due to the macrophage-derived TNF-α (113). Additionally, in COVID-19 patients, serum levels of IL-6, TNF-α, IL-8, and IL-10 were negatively correlated with lymphocyte counts (114).

Collectively, neutrophilia, lymphopenia, dysregulated monocyte and macrophages, cytokine storm, and coagulation disorders lead to organ failure and other COVID-19-associated disorders, which may be exacerbated in the elderly due to the inflammatory conditions of these disorders following SARS-CoV-2 infection.

### 3.2 Role of pro-inflammatory cytokines

In accordance with the knowledge of coronaviruses, it appears that cytokines and chemokines play a pivotal role in the immunopathogenesis of SARS-CoV, MERS, and SARS-CoV-2. An imbalanced production of these mediators causes hyperinflammation and damage to various organs, including the lungs, liver, kidneys, and heart (113). There is a possibility that some COVID-19-infected patients develop severe symptoms, resulting in hyperinflammation caused by cytokines/chemokines overexpression, a pathologic condition known as cytokine release syndrome (CRS), which can lead to pneumonia and ARDS (115). A series of immune responses trigger a systemic CRS after the virus invades the respiratory mucosa and infects various immune and non-immune cells, such as alveolar type II (116). CRS usually manifests as a systemic inflammatory immune response associated with the elevation of inflammatory biomarkers and multiple organ failure due to an intensified release of pro-inflammatory cytokines (117, 118). Tissue damage by SARS-CoV-2 infection or an overactivated immune system can cause CRS. A sign of this overactive immune system is the infiltration of macrophages and neutrophils in the affected tissues. These cells migrate to the infected tissue to provide protection, but their uncontrolled functions cause hyperinflammation and tissue damage (119). Although numerous cytokines and chemokines are involved in the pathogenesis of SARS-CoV-2, the most significant inflammatory cytokines, including TNF-α, IL-1β, IL-6, IL-8, and IL-17 that cause tissue damage and multi-organ failure, have been briefly discussed here (**Table 1**).

**Table 1:** Role of the most studied pro-inflammatory cytokines in the pathogenesis of COVID-19.

Cytokine	Source	Inducer	Functions in COVID-19	Ref
**TNF-α**	➢ Epithelial cells ➢ Smooth muscle cells ➢ Alveolar macrophages ➢ T cells ➢ Mast cells	➢ IL-17 ➢ IL-1β	➢ Participating in CRS ➢ Inducing the expression of IL-1β and IL-6 ➢ Inducing T cell apoptosis ➢ Neutrophil-mediated bronchial hyperresponsiveness and airway inflammation ➢ Increasing disease severity ➢ Decreasing the number of T cells	(11) (120) (9, 121) (122)
**IL-1β**	➢ B cells ➢ Neutrophils ➢ DCs ➢ Monocytes ➢ Macrophages	➢ Inflammasomes ➢ TNF-α	➢ Inducing the expression of chemokines, adhesion molecules, IL-6, iNOS, PLA, and COX2 ➢ Inducing vasodilation, hypotension, pain, fever, hyperinflammation, and migration of immune cells into the site of infection ➢ Inducing tissue damage ➢ Pyroptosis ➢ Stimulating the production of pro-inflammatory cytokines ➢ Vascular leakage	(123) (124–126) (127)
**IL-6**	➢ Lung epithelium ➢ Monocytes ➢ Macrophages ➢ DCs ➢ Lymphocytes ➢ Fibroblasts	➢ IL-1β ➢ TNF-α	➢ Inducing hematopoiesis, B-cell differentiation, platelet generation, coagulation, and inflammation ➢ Participating in CRS ➢ Stimulate hepatocytes to produce acute-phase proteins ➢ Decreasing the number and function of T cells ➢ Increasing the frequency of exhausted T cells	(128) (129) (111) (117)
**IL-8**	➢ Epithelial cells ➢ Endothelial cells ➢ T cells	➢ IL-1β ➢ IL-9 ➢ IL-12 ➢ IL-17 ➢ TNF-α	➢ Recruiting and activating neutrophils ➢ Inducing NETosis ➢ Participating in CRS ➢ Inducing angiogenesis ➢ Inducing ROS production ➢ Increasing disease severity ➢ Increasing Cit-H3, cfDNA, and MPO-DNA levels ➢ Inducing multi-organ damage	(118) (130, 131) (9, 132, 133) (134)
**IL-17**	➢ Th17	➢ IL-1β ➢ IL-12 ➢ IL-15 ➢ IL-23	➢ Inducing the production of IL-6, IL-8 ➢ Recruiting neutrophils ➢ Increasing inflammatory responses ➢ Inducing thrombosis ➢ Participating in CRS ➢ Supporting virus replication and persistence ➢ Preventing apoptosis of virus-infected cells ➢ Inducing lung lesions and ARDS ➢ Tissue damage	(135, 136) (137) (138) (139)

#### 3.2.1 Tumor necrosis factor-α

It has been demonstrated that TNF-α is a crucial factor in the pathophysiology of CRS in patients with SARS-CoV-2 infection. Several types of cells in the airways are responsible for TNF-α production, including epithelial cells, smooth muscle cells, alveolar macrophages, T cells, and mast cells. The synthesis of this cytokine is primarily triggered by PAMPs and IL-1β following NF-κB activation. IL-17 also induces the expression and release of TNF-α (140). Interestingly, TNF-α induces the expression of IL-1β and IL-6 as well as T cell apoptosis (12). It has been reported that inhaling TNF-α leads to neutrophil-mediated bronchial hyperresponsiveness and airway inflammation in healthy individuals (120). Recent studies have shown that serum levels of TNF-α have increased significantly in patients with COVID-19, and there is a positive and significant association between elevated serum TNF-α levels and disease severity (10, 130). In contrast, increasing TNF-α levels are negatively associated with the number of T cells because, as discussed, this cytokine can induce T-cell apoptosis (131).

#### 3.2.2 Interleukin-1β

IL-1β is a pro-inflammatory pleiotropic cytokine that plays a pivotal role in inflammatory-based disorders. B cells, neutrophils, DCs, monocytes, macrophages, and synovial fibroblasts can produce IL-1β (122). Moreover, IL-1β induces the expression of chemokines, adhesion molecules, IL-6, inducible nitric oxide synthase (iNOS), phospholipase A (PLA), and type 2 cyclooxygenase (COX2). The release of the mentioned mediators leads to vasodilation, hypotension, pain, fever, hyperinflammation, and migration of immune cells into the site of infection, inducing tissue damage (122–124). Recent studies have reported a significant increase in the expression of IL-1β at the mRNA and protein levels in patients with COVID-19 (10, 141).

Moreover, inflammasomes play a significant role in the production of IL-1β. For example, the activation of NOD-, LRR- and pyrin domain-containing protein 3 (NLRP3) activates mechanisms related to pyroptosis, inflammatory-based programmed cell death, resulting in IL-1β release by the pyroptotic cell, which is a common phenomenon in cytopathic viral infections (138). A crucial role of pyroptosis in SARS-CoV-2 infection is stimulating the production of pro-inflammatory cytokines, which lead to destructive inflammatory responses and tissue damage. It has previously been observed in patients with SARS-CoV infection that viral infection and replication could cause high levels of virus-induced pyroptosis and vascular leakage (142). Therefore, IL-1β should be considered a critical immune mediator in the pathogenesis of COVID-19.

#### 3.2.3 Interleukin-6

Another pro-inflammatory and pleiotropic cytokine is IL-6, expressed by several cells, including monocytes, macrophages, DCs, lymphocytes, and fibroblasts (125). The circulatory levels of IL-6 increase in several inflammatory states, such as viral infections, septic shock, burns, and trauma (125). IL-6 is an immune system regulator involved in hematopoiesis, B-cell differentiation, platelet generation, coagulation, and inflammation. This cytokine also significantly participates in CRS (117). Another critical function of IL-6 is stimulating hepatocytes to produce acute phase proteins such as C-reactive protein (CRP). A significant increase in this protein has been reported in COVID-19 patients (141). In patients with moderate or severe COVID-19, serum levels of IL-6 are significantly elevated. However, some studies reported that circulatory concentrations of IL-6 were associated with disease severity (143–145). Although the origin of this increase in serum level is probably not circulating blood cells like peripheral blood mononuclear cells (PBMCs) and is more related to the lung epithelium. Studies have shown that IL-6 transcript levels in COVID-19 patients did not change significantly compared to controls that confirm this theory (146). Like TNF-α, an increased level of IL-6 is associated with a decrease in the number and function of T cells. Furthermore, in COVID-19 patients with increased levels of IL-6, the frequency of exhausted T cells is elevated (147).

#### 3.2.4 Interleukin-8

IL-8, or CXCL8, is a chemoattractant and priming factor for neutrophils and is also involved in inflammatory responses, NETosis (neutrophil extracellular trap [NET]), and angiogenesis (132). In addition, IL-8 activates neutrophils to produce inflammatory mediators and clearance of bacterial infections by inducing reactive oxygen species (ROS) production, as well as NET formation (128, 129). IL-8 levels significantly increase in patients with SARS-CoV-2 infection and are directly related to the disease severity (10, 134, 148). Following the formation of NETosis in patients with COVID-19, citrullinated histone H3 (Cit-H3), cell-free DNA (cfDNA), and myeloperoxidase (MPO)-DNA levels are increased, inducing multi-organ damage and death from severe SARS-CoV-2 infection (149). Since neutrophils are one of the main factors in the pathogenesis of COVID-19, IL-8, as a chemotactic factor of these cells, can play an essential role in increasing neutrophil-mediated destructive inflammatory responses.

#### 3.2.5 Interleukin-17

The IL-17 family comprises six members (A-F), which IL-17A and IL-17F types are involved in inflammation (150). This cytokine is secreted from Th17 cells and can induce the production of IL-6, IL-8, and several chemokines in response to viral infections, recruiting neutrophils and increasing inflammatory responses (151, 152). The systemic effects of IL-17 can be explained by inducing coagulation pathways and coagulopathy, as well as CRS (153, 154). Evidence demonstrated that IL-17 and TNF-α could create a synergistic effect in initiating these processes. Microthrombosis can damage various organs during and after SARS-CoV-2 infection (135).

Moreover, IL-17, along with IL-6, can support virus replication and persistence, as well as prevent apoptosis of virus-infected cells (136). IL-17 concentrations significantly increased in COVID-19 patients, possibly associated with lung lesions and ARDS (155). The analysis of biopsy samples taken from the liver, lung, and heart tissue of patients who died of severe SARS-CoV-2 infection has shown that the infiltration of Th17 and CD8^+^ T cells in the lung tissue of these people has increased significantly (156). These findings indicate that the dysregulated increase in the activity of Th17 and cytotoxic CD8^+^ T cells has led to severe tissue damage and, finally, the death of patients.

The reviewed studies indicate that the excessive increase in pro-inflammatory cytokines during SARS-CoV-2 infection can cause tissue damage through different mechanisms. However, the patterns of these cytokines at different ages are probably different. Therefore, targeting these cytokines or their receptors can be a potential treatment strategy. However, more studies are required because inhibiting cytokines can act like a double-edged sword and increase viral load or co-infections.

## 4 COVID-19-mediated liver injury

**Figure 2:**
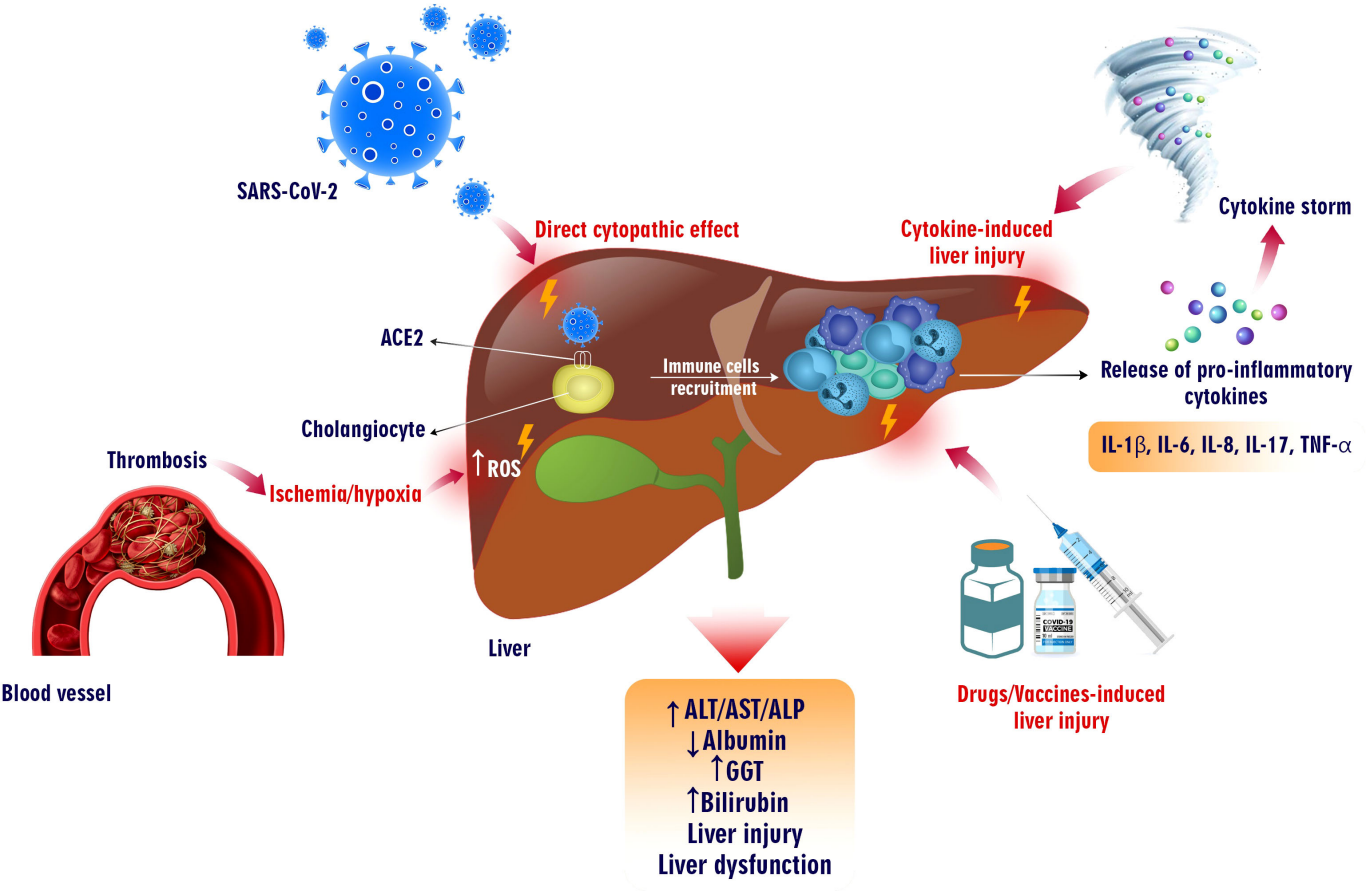
COVID-19-induced liver injury. SARS-CoV-2 infection can cause liver failure in different ways. This virus can directly infect liver cells expressing ACE2 and exert cytopathic effects. Additionally, the increase in the production of pro-inflammatory cytokines from virus-infected cells has led to the recruiting of immune cells into the liver, which can cause a cytokine storm and tissue injury. Moreover, thrombosis causing hypoxia and ischemia induces ROS overproduction, which harms liver tissue. On the other hand, vaccinations and drugs prescribed for COVID-19 can also increase liver enzymes, bilirubin, GGT, and various degrees of liver damage.

Although SARS-CoV mainly affects the respiratory system, it can also lead to systemic and organ-specific disorders. During the earlier SARS-CoV-2 outbreak, about 60% of patients experienced varying degrees of liver impairment. According to evolutionary similarities, it is conceivable that SARS-CoV-2 also damages the liver with hepatobiliary manifestation and enzyme elevation. Patients with COVID-19 may have pre-existing liver disease or not (157, 158). A recent systematic review and meta-analysis reported that 14-53% of COVID-19 patients suffer from varying degrees (mild to severe conditions) of liver injury (159). However, the mechanism of liver injury has not been fully discovered. It might be due to several underlying mechanisms, including severe inflammatory responses, direct cytopathic effects, aggravation of the pre-existing liver disease, and cytokine storm. Moreover, drug-induced liver injury can occur in undertreatment patients, as explained in the next part (**Figure 2**) (137, 160).

SARS-CoV-2 can damage the liver directly through ACE2, an entry receptor located on bile duct cells (cholangiocytes) (59.7%) and less on hepatocytes (2.6%), leading to hepatocyte apoptosis (161). However, recent data revealed that other causative factors, such as drug-induced injury, precede direct cytotoxicity (162). Liver biopsies of COVID-19 patients showed lobular inflammation, acidophilic bodies, mild activity of the portal vein, and moderate microvascular steatosis. Moreover, autopsies from dead patients revealed hepatomegaly, lobular necrosis, slight lymphocyte infiltration, and sinusoidal dilation of the central lobule in at least 50% of patients. It is unknown if the viral infection or the medications are responsible for these histopathological changes (158, 163, 164).

The severity of COVID-19 elevated IL-6 or ferritin levels and younger age are considered strong risk factors for liver injury during hospitalization and are associated with mortality (160, 165). The mitochondrial alterations and metabolic acidosis aggravation are related to SARS-CoV-2 infection and result in ischemic/hypoxic liver injury (166). Several studies revealed that patients had abnormal liver function tests, including a moderate increase in bilirubin, aspartate aminotransferase (AST), and alanine aminotransferase (ALT), which are frequently observed in patients with severe COVID-19 compared with non-severe patients (167–169). The alteration of these biomarkers correlates with liver function, and hepatocyte integrity is involved in acute liver infection and disease severity. Besides, the transaminase increase could be associated with morbidity and mortality (170).

However, hypoalbuminemia has been reported due to increased capillary permeability and decreased hepatic synthesis in severe patients. Elevated alkaline phosphatase (ALP) and GGT levels are observed in acute inflammatory oxidative stress and are less common in COVID-19 (26, 171). Interestingly, skin darkening and hyperpigmentation caused by increased estrogen and alteration of tyrosine into melanin in patients after recovery are recognized because of liver injury (172).

It has been revealed that patients with pre-existing liver disease and systematic inflammation are more susceptible to COVID-19-mediated liver injury (173). Comorbidities such as metabolic-associated fatty liver disease (MAFLD), alcohol-related liver disease, autoimmune hepatitis (AIH), and chronic liver disease (CLD) aggravate COVID-19 severity (171). In patients with fatty liver disease, alcohol use disorder (AUD), and non-alcoholic fatty liver disease (NAFLD), GGT levels were elevated compared with those without pre-existing liver disease, indicating that these patients are more prone to severe disease phenotype (162, 170).

### 4.1 Impacts of aging

Several studies have investigated the association between age and severity of liver injury in COVID-19 patients. A retrospective study comprising 900 patients classified for ages 18-39, 40-69, and more than 70 years old demonstrated that comorbidity and C-reactive peptide (CRP) are positively associated with aging. Moreover, abnormalities in liver function tests and liver-related mortality were observed, especially in 40-69 years old patients. Additionally, they had higher ALT levels than the other groups (174).

Another study reported that hepatic symptoms are more frequent in young and obese patients with pre-existing liver diseases like MAFLD. These symptoms are aggressive in adult patients having severe COVID-19 (175). With advanced age and increased comorbidities, such as arterial hypertension, diabetes mellitus, chronic liver, cardiovascular disease, and malignancy, the risk of liver injury and mortality increases in elderly patients (176). A retrospective cohort study revealed that COVID-19-associated liver injury is more common in patients aged ≥ 65 years, especially with other comorbidities. Elevated levels of AST and total bilirubin were indicators of increased risk of mortality (177).

Some studies agree that COVID-19 patients under 18 suffer less from liver injury than adults (17, 178). A retrospective case series study reported that two COVID-19 patients under one year without previous liver disease presented with acute liver failure, and three patients aged 2, 8, and 13 exhibited hepatitis with cholestasis in liver biopsies (179). Indeed, liver injury in children with COVID-19 is mild, with low changes in laboratory and radiological evidence (180). Taken together, there seems to be a positive association between aging and liver injuries, particularly regarding pre-existing liver involvement.

### 4.2 Role of cytokines

Systemic homeostasis disruptions in both vascular and immune-mediated pathways affect multiple mechanisms in the liver. Some studies have demonstrated that COVID-19 patients have minor early symptoms but might develop quickly into the final stage of multiple organ dysfunction (22). This trend is associated with a viral infection-driven rapid onset of inflammatory cytokines. As discussed, dysregulated activation of inflammatory pathways leads to the hyperproduction of cytokines and, eventually, the formation of a cytokine storm (181). The overexpression of CRP, ferritin, LDH, TNF-α, IL-1β, IL-6, IL-2, IFN-γ, and vascular endothelial growth factor (VEGF) can be associated with COVID-19 severity (160, 166, 182). It has been reported that there is no difference between cytokine profiles in mild or severe COVID-19, ARDS, and sepsis. However, the therapeutic regime in various cytokine storm-induced disorders differs (183). Several studies suggested that pro-inflammatory cytokines contribute to hepatologic abnormalities in the early and late phases (171, 184, 185). A large cohort study demonstrated a correlation between systematic inflammation and the acute phase of liver injury in which IL-6 is accompanied by CRP or ferritin-caused elevation in AST levels (186). The increase in ALP and GGT levels is related to the advanced phase of liver injury, which is mediated by cytokines and causes hepatocellular cholestasis (187). Moreover, SARS-CoV-2, by activating twenty family member 2 and C-type lectins on myeloid cells, strengthened pro-inflammatory cytokine responses (170). In inflammatory states, peripheral blood evaluation revealed hyperactivation of CD4^+^ T cells, particularly CCR6^+^ Th17 cells, and CD8^+^ cytotoxic T cells, inducing hepatocellular dysfunction (188).

The pathogenic effect of cytokines on liver injury caused by COVID-19 is not well known. However, according to the evidence, some pathophysiological prosses can be suggested that are associated with hepatocellular damage. Cytokine can induce hypoperfusion, vascular dysfunction, and oxidative stress. Moreover, these mediators can cause vascular permeability by increasing the adherence of immune cells to the endothelial (22). Both vascular permeability and ROS propagation result from cytokine activation (22, 189). For instance, TNF-α can impair NO-mediated vasodilation, and capillary leak syndrome and hypoxia occur dramatically with the continuation of this process. Capillary leakage causes edema in some organs, such as the liver, brain, heart, and kidneys. Upregulation of IFN-related JAK-STAT signalling in liver autopsies exhibited vascular damage, endothelial injury, and recruiting immune cells, leading to clot formation (190). Hepatocellular injury can activate Kupffer cells to produce ROS, NO, and proinflammatory cytokines. These products induce liver sinusoidal endothelial cells (LSECs) to respond to the cytokines that cause more liver damage (191). Under the influence of TNF, hepatocytes secrete IL-6, which activates caspase 3 and hepatocellular apoptosis. Moreover, the generated NO causes mitochondrial damage and hepatocellular necrosis (192).

SARS-CoV-2, through ACE2 and upregulation of angiotensin II (Ang II), plays a crucial role in RAS signalling, promoting inflammation, tissue injury, and migration of endothelial cells (193). hypercytokinemia is involved in the renin-angiotensin-aldosterone system. Therefore, the imbalance of this system by inducing the accumulation of bradykinin and plasminogen activator inhibitor-I leads to thrombosis (22). The consequence of this circumstance is inflammation in the liver and hepatocyte injury (194).

## 5 Effects of COVID-19 therapeutic regimen and vaccination on liver injury

The COVID-19 pandemic has caused widespread morbidity and mortality. This virus can affect numerous organs such as the lung, kidney, heart, CNS, and liver causing multiorgan failures. As a result, there is an urgent need for an effective method to limit the spread of the COVID-19 virus, and vaccine development may be a promising option (195). Although adverse effects from vaccination have been minor, there are also reports of liver damage following vaccination with different platforms of the COVID-19 vaccine (**Table 2**) (217).

**Table 2:** Impacts of drugs/vaccines on liver injury in patients with COVID-19.

	Generic name	Function/ Dose	Outcomes	Type of study	Ref/NCT
**Drug**	**Remdesivir**	Antiviral (RdRp inhibitor) (viral replication inhibitor)	↑ ALT and AST ↑ ALT and AST and bilirubin ↑ ALT and AST Hyper transaminasemia and ↑ bilirubin ↑ ALT and AST ↑ ALT, AST and bilirubin ↑ ALT and AST ↑ ALT ↑ ALT and AST ↑ ALT and AST ↑ ALT, AST and bilirubin	Clinical trial Randomized Controlled Trial Clinical Trial Clinical Trial Case Report Clinical Trial Case Reports Case Reports Case Series Clinical Trial Case Series	(196), NCT04280705 (197), NCT04257656 (198), NCT04292730 (199) (200) (201), NCT04292899 (202) (203, 204) (205) (206) (207)
	**Lopinavir/ritonavir**	Antiviral (Protease inhibitor) (Viral replication inhibitor)	↑ ALT and AST ↑ ALT ↑ ALT, AST, GGT and bilirubin ↑ ALT, AST and bilirubin ↑ ALT, AST, ALP and bilirubin	Randomized Controlled Trial Randomized Controlled Trial Retrospective Single-Center Study Retrospective Analysis Retrospective Analysis	(208), ChiCTR2000029308 (209), NCT04252885 (210) (211) (212)
	**Favipiravir**	Antiviral (RdRp inhibitor)	↑ AST ↑ ALT, AST, ALP, GGT and bilirubin	Randomized Controlled Trial Case Reports	(213), ChiCTR2000030254 (214)
	**Hydroxychloroquine**	Antiparasitic Agents	↑ AST ↑ ALT and AST ↑ Liver function test	Randomized controlled trial Case Report Retrospective Analysis	(215), NCT04356937 (216) (217)
	**Tocilizumab**	IL-6R antagonist	↑ ALT and AST ↑ ALT and AST ↑ ALT, AST and GGT	Randomized Controlled Trial Case Reports Case Reports	(218), NCT04356937 (210) (219)
**Vaccine**	**Pfizer BioNTech**	1^st^ dose 2^nd^ 2^nd^ 1^st^ 1^st^ and 2^nd^ 2^nd^ 1^st^ -	↑ ALT, AST, ALP and Bilirubin ANA and Anti ds-DNA positive ↑ ALT, AST, ALP, GGT and Bilirubin ANA positive ↑ ALT, ALP and Bilirubin ↑ ALT, ALP and Bilirubin Anti-ds-DNA positive ↑ ALT and AST ANA positive ↑ ALT and AST ANA and ASMA positive ↑ ALT, AST and Bilirubin ↑ ALT, AST, GGT and Bilirubin	Case Reports Case Reports Case Reports Case Series Case Series Case Reports Case Reports Case Reports	(190) (191) (220) (215) (221) (222) (223) (224)
	**ChAdOx1 nCoV-19 vaccine (Oxford AstraZeneca)**	1^st^ 1^st^ 1^st^	↑ ALT, AST, ALP, GGT and Bilirubin ANA positive ↑ ALT and AST ↑ ALT, AST and bilirubin ANA and ASMA positive	Case Reports Case Reports Case Reports	(225) (226) (227)
	**SARS-CoV-2 Moderna vaccine (mRNA-1273)**	2^nd^ 1^st^ 1^st^ 1^st^ 1^st^ 1^st^ 1^st^ 1^st^ 1^st^	↑ ALT, AST, ALP, GGT and Bilirubin ANA, ASMA, ASLA positive ↑ ALT, ALP and Bilirubin ASMA positive ↑ ALT, AST and ALP ANA and ASMA positive ↑ ALT, AST and Bilirubin ANA and Anti ds-DNA positive ↑ ALT, AST and Bilirubin ↑ ALT, AST and Bilirubin ANA, ASMA positive ↑ ALT and Bilirubin ↑ ALT, AST and Bilirubin ↑ ALT, AST and Bilirubin ANA positive	Case Reports Case Series Case Reports Case Reports Case Reports Case Reports Case Reports Case Reports Case Reports	(228) (229) (230) (222) (231) (232) (233) (234) (235)
	**Sinopharm**	2^nd^	↑ ALT, AST and Bilirubin	Case Reports	(203)

↑ means "increase".

### 5.1 Effect of vaccination

Several COVID-19 vaccine platforms have been effective in preventing SARS-CoV-2 infection. Despite mild side effects, no evidence of acute liver injury (ALI) has been reported among vaccine recipients, possibly due to the small sample size or other involved factors. Since the extensive implementation of COVID-19 immunization programs worldwide, reports of adverse events have been reported, including rare hepatic adverse effects with various characteristics in the form of ALI resembling autoimmune hepatitis (AIH). Since the widespread implementation of COVID-19 immunization programs worldwide, reports of adverse events have been made, including rare hepatic adverse effects with various characteristics in the form of ALI resembling autoimmune hepatitis (AIH) (206, 230, 236). The exact mechanism of liver injury caused by the COVID-19 vaccine is unknown. Therefore, there are no data to prove the existence of a relationship between vaccination and the incidence of AIH (237).

Vaccine-induced AIH was reported in both mRNA and non‐mRNA vaccines, suggesting that these adverse effects could be independent of the vaccine mechanisms (208). Some patients had a liver or autoimmune disease history, whereas others had neither liver nor autoimmune disease history (211).

The pattern of liver injury reported was predominantly hepatocellular, and some patients showed features of immune-mediated hepatitis (214). In some patients, liver injury was observed after the first vaccination dose, whereas in others, it occurred after the second dose (214). Elevated bilirubin levels and liver enzymes, which clinically manifest as jaundice due to hepatotoxicity, are examples of abnormal liver function tests (230, 236). The most frequently reported symptom was jaundice (211). [249] The seropositivity rate for anti-smooth muscle antibody (ASMA), anti-nuclear antibody (ANA), and anti-mitochondrial antibody (AMA) varied between studies, and the IgG level was high in most of the ¬patients (214). Liver biopsies exhibited a varied pattern, including histological features consistent with AIH, such as interface hepatitis, portal inflammation, and non-specific centrilobular necrosis (228). Additionally, after vaccination, severe thrombosis in the portal and splenic veins and immune thrombocytopenic purpura were linked with ALI (218, 220).

A case series described two patients with hepatocellular liver injury without autoimmune features on serology and histology following COVID-19 vaccination (215). However, another case series analysis showed no evidence of ALI after COVID-19 vaccination (217).

### 5.2 Effect of therapeutic regimen

Drug-induced liver injury (DILI) is a rare and potentially life-threatening adverse effect seen with different chemicals or drugs (224). Some liver abnormalities observed in COVID-19 are associated with DILI manifested by high liver enzyme levels and, less commonly, high bilirubin levels. Patients’ histology showed moderate microvascular steatosis and minor hepatic inflammation, possibly due to DILI from treating the virus or its symptoms. DILI in patients with COVID-19 is a mild hepatic inflammation that occurs following antiviral treatments or the disease itself. Histological examination shows moderate microvascular steatosis, manifested by elevated liver enzymes and rarely bilirubin (158, 205).

Antiviral (remdesivir, favipiravir, lopinavir/ritonavir, and umifenovir), antibiotics (azithromycin), immunomodulators (dexamethasone and tocilizumab), antimalarial (hydroxychloroquine), and antipyretic medications (acetaminophen) have been administred to patients with COVID-19 (225, 227). Several of the mentioned medications have previously been linked to various degrees of hepatotoxicity when used to treat other disorders, such as viral infections. The administration of many of these drugs in similar viral infections is associated with any grade of hepatotoxicity.

Almost all medications used to treat COVID-19 are metabolized in the liver, but some degree of liver damage is unavoidable (229). However, it is unknown what causes the elevated liver enzymes in this population (the disease or DILI). Additionally, due to drug combinations, it is challenging to imagine a relationship between each drug and liver damage (232).

Remdesivir is a nucleoside analog currently approved by the United States food and drug administration (FDA) and recommended for some hospitalized patients with COVID-19 (233). This antiviral drug shortens recovery time in hospitalized patients with lower respiratory tract SARS-CoV-2 infection. Despite its efficacy in reducing recovery times, its healing properties are yet to be proven (238). Increasing ALT/AST, hyperbilirubinemia, and hypoalbuminemia are considered the most common adverse effects in patients under treatment with remdesivir (197, 234, 239).

The efficacy of Umifenovir in COVID-19 patients was assessed in a randomized clinical trial and showed significantly improved clinical and laboratory parameters and decreased hospitalization duration; however, the most common liver-associated disorder in patients using umifenovir was abnormal liver function tests (235).

LPV/r is a co-formulation of ritonavir and lopinavir structurally related protease inhibitors (203). Conflicting published data results have sparked debate about using LPV/r in COVID-19 patients. Two clinical trials showed that a combination of LPV and ritonavir made no difference to the standard of care. However, some studies have reported that LPV/r could decrease the SARS-CoV-2 shedding and the median time of clinical improvement (240–242). Abnormal liver function has been reported in patients under treatment with lopinavir/ritonavir, and the most prevalent increase in test findings was seen in GGT and total bilirubin (205, 243).

As an RNA polymerase inhibitor, favipiravir reduces viral clearance time and improves chest imaging. However, its administration could be associated with adverse effects, such as increased liver enzymes (244, 245).

The liver is the primary site of azithromycin metabolism, and prior studies found that azithromycin had a modest risk of acute, temporary, and asymptomatic increases in serum aminotransferases, occurring in up to 2% of individuals with incomplete treatment. Thus, when given to COVID-19 patients, especially in small doses and for a brief time, this medication can be excluded from further evaluation associated with liver injury (231, 246).

Patients with severe COVID-19 have a high level of circulating IL-2, IL-6, IL-7, IL-10, and IFN-γ (10). Therefore, immunomodulator drugs could help regulate inflammatory responses, enhancing COVID-19 prognosis (224). Dexamethasone is the first drug shown to reduce mortality in patients with severe COVID-19 (247). This drug is metabolized in the liver and has little effect on hepatic dysfunction in COVID-19 patients because of the low prescribed dosage and short treatment duration (248). Additionally, tocilizumab is a recombinant humanized monoclonal antibody that inhibits the IL-6 receptor and has been used to reduce IL-6-mediated inflammatory responses in patients with severe COVID-19. Patients receiving tocilizumab had a better median overall survival than hospitalization time. A retrospective study of patients treated with tocilizumab showed no evidence of adverse liver effects (249). Other studies reported DILI after using tocilizumab and mild to moderate elevations in liver enzyme levels (219, 221).

SARS-CoV-2 infects ACE2-expressing cells, and chloroquine may inhibit the ligation of the virus to the ACE2 by inhibiting terminal glycosylation (250). The effectiveness of hydroxychloroquine/chloroquine is controversial in patients with COVID-19 (251, 252). It has been reported that hydroxychloroquine could be a probable but uncommon cause of DILI (246). A clinical study demonstrated that patients taking hydroxychloroquine alone or along with azithromycin had higher liver enzymes (213). Furthermore, a case study showed hepatotoxicity and transaminase elevation after using hydroxychloroquine (253). Acetaminophen has been the medication for treating fever and myalgia associated with COVID-19 (254), and using acetaminophen in supratherapeutic amounts for several days might lead to hepatitis, cholestasis, or other nonspecific elevations of liver enzymes (255).

JAK inhibitors, such as baricitinib, imatinib, and tofacitinib, help reduce mortality and intubation rates. These inhibitors have been approved in several countries to treat COVID-19. However, administering JAK inhibitors could be associated with increased liver enzymes and bilirubin in less than 1% of patients with COVID-19. Furthermore, no severe DILI cases have been reported following treatment with JAK inhibitors (256).

## 6 Conclusion

According to the available studies on COVID-19, liver dysfunction generally develops in male, obese, and elderly patients. Several mechanisms are involved in liver injury, including severe inflammatory response, a direct cytopathic effect, and the idiosyncratic effect of therapeutic regimens and cytokine storm. Mild to severe liver injury is frequent and occurs in approximately 50% of patients with COVID-19 and is associated with the development of the disease, particularly in the elderly with pre-existing liver disease and other comorbidities. The patients hospitalized due to the worsening of the disease have a significant elevation in serum levels of AST, ALT, and ALP and reduced albumin levels. Elevations in liver enzymes are transient and decrease after recovery. Therefore, due to the changed conditions of the immune system and chronic inflammation caused by aging, the elderly should be under more care for managing tissue damage, especially the lung, liver and heart, because the increased levels of pro-inflammatory cytokines or the prescribed drugs worsen the condition of these patients. Furthermore, post-vaccination liver complications should be monitored in the elderly.

## Author contributions

All authors participated in drafting the manuscript. RF and RE supervised the study and reviewed the final version of the manuscript. All authors contributed to the article and approved the submitted version.
